# Supplementary material for: Lung Cancer Incidence by Detailed Race–Ethnicity
Source: Cancers (Basel). 2023 Apr 5;15(7):2164. doi: 10.3390/cancers15072164 (PMC10093016; doi:10.3390/cancers15072164)
Supplement: Supplementary file 1 [file cancers-15-02164-s001.zip › cancers-2265161-supplementary.pdf]

Supplementary Table S1. Age-Adjusted Incidence Rates of Lung Cancer (Overall, Histologically Confirmed Adenocarcinoma, Distant SEER Stage at Diagnosis, and Microscopically Verified Cases) by Detailed Race-Ethnicity and Sex. Florida, 2012–2018.

Race-Ethnicity	Overall Incidence Rate[b] (95% CI)		Adenocarcinoma Histology Incidence Rate[b] (95% CI)		Distant SEER Stage at Diagnosis Incidence Rate[b] (95% CI)		Microscopically Verified Incidence Rate[b] (95% CI)	
	**Male**	**Female[a]**	**Male**	**Female[a]**	**Male**	**Female[a]**	**Male**	**Female[a]**
**Non-Hispanic White**	71.4 (70.8-72.1)	61.0 (60.4-61.6)	27.2 (26.8-27.6)	28.4 (28.0-28.8)	33.7 (33.3-34.2)	27.0 (26.6-27.4)	64.6 (64.0-65.2)	56.3 (55.7-56.8)
**Non-Hispanic Black**	63.5 (61.8-65.3)	36.0 (34.9-37.1)	24.9 (23.8-26.0)	17.3 (16.6-18.1)	32.6 (31.3-33.8)	16.7 (15.9-17.4)	57.2 (55.5-58.9)	33.1 (32.0-34.2)
US Born Black	81.7 (79.3-84.2)	46.4 (44.8-48.0)	30.7 (29.3-32.2)	21.6 (20.6-22.7)	41.6 (39.9-43.4)	21.3 (20.3-22.4)	73.3 (71.1-75.7)	42.7 (41.2-44.2)
Caribbean-Born Black	26.2 (24.1-28.4)	14.9 (13.6-16.3)	12.7 (11.3-14.3)	8.7 (7.7-9.8)	13.7 (12.3-15.3)	7.2 (6.3-8.2)	23.9 (21.9-26.0)	13.2 (12.0-14.5)
**Non-Hispanic API**	25.5 (23.2-27.9)	18.8 (17.2-20.6)	13.9 (12.3-15.7)	12.8 (11.4-14.2)	14.3 (12.6-16.1)	11.1 (9.8-12.5)	24.3 (22.1-26.6)	18.2 (16.6-19.9)
**Hispanic**	49.8 (48.6-51.0)	26.4 (25.7-27.1)	20.4 (19.6-21.1)	13.2 (12.7-13.8)	23.3 (22.5-24.1)	11.7 (11.2-12.2)	45.0 (43.8-46.1)	23.8 (23.1-24.5)
Mexican	36.8 (31.7-42.4)	16.8 (14.0-19.8)	13.2 (10.3-16.5)	8.2 (6.4-10.4)	18.2 (14.8-22.0)	9.9 (7.8-12.2)	32.0 (27.3-37.1)	13.7 (11.3-16.5)
US Born Mexican	59.8 (49.5-71.3)	23.5 (18.5-29.2)	22.3 (16.4-29.4)	12.9 (9.4-17.1)	28.5 (21.7-36.4)	13.3 (9.8-17.6)	52.9 (43.4-63.5)	21.1 (16.4-26.4)
Foreign-Born Mexican	22.3 (17.4-28.2)	12.2 (9.1-16.0)	7.4 (4.8-10.9)	5.1 (3.2-7.7)	11.7 (8.5-15.8)	7.8 (5.3-11.0)	18.9 (14.5-24.2)	8.6 (6.2-11.8)
Puerto Rican	43.7 (41.2-46.4)	28.8 (27.1-30.7)	17.9 (16.3-19.6)	13.2 (12.0-14.5)	21.2 (19.5-23.0)	13.2 (12.0-14.4)	39.8 (37.4-42.3)	26.2 (24.5-28.0)
Cuban	65.6 (63.6-67.6)	31.7 (30.5-33.0)	25.3 (24.1-26.6)	15.5 (14.6-16.4)	30.1 (28.7-31.4)	13.8 (12.9-14.6)	59.5 (57.6-61.4)	28.8 (27.6-30.0)
Dominican	44.2 (37.8-51.2)	25.2 (21.8-29.1)	20.9 (16.7-25.8)	14.6 (12.0-17.6)	19.8 (15.7-24.6)	10.4 (8.2-13.0)	38.7 (32.9-45.2)	22.4 (19.1-26.0)
Central and South American	30.8 (28.8-32.9)	20.8 (19.5-22.2)	15.3 (13.9-16.8)	11.4 (10.5-12.4)	15.9 (14.4-17.4)	9.8 (8.9-10.7)	27.9 (26.0-29.9)	18.6 (17.4-19.9)
Central American	24.1 (20.6-28.1)	16.3 (14.3-18.5)	10.6 (8.3-13.2)	8.0 (6.6-9.5)	13.0 (10.4-15.9)	8.5 (7.0-10.1)	21.6 (18.3-25.3)	14.6 (12.7-16.7)
South American	33.4 (31.0-36.0)	22.9 (21.3-24.6)	17.2 (15.5-19.1)	13 (11.8-14.3)	17.1 (15.4-18.9)	10.4 (9.3-11.6)	30.3 (28.1-32.8)	20.4 (18.9-22.0)

a. Includes n=39 identified as non-binary; b. Rates are annual, per 100,000, and age adjusted to the U.S. 2000 Standard Population.
Abbreviation: CI, Confidence Interval; US, United States of America; API, Asian/Pacific Islander.
